# Why do people post when they or others are under risk or threat? Sociological and psychological reasons

**DOI:** 10.3389/fpsyg.2023.1191631

**Published:** 2023-12-05

**Authors:** Fatih Yaman

**Affiliations:** Muş Alparslan University, Muş, Türkiye

**Keywords:** posts, social media, social media posts, psychological reasons for posting, sociological reasons for posting

## Abstract

**Introduction:**

Advances in technology make it easier for users to post content on social media. People can post different types of content in digital environments. Sometimes, they post such content in risky situations. Accordingly, this study aims to determine the sociological and psychological reasons why people record dangerous occurrences where they or other people are under risk or threat and post these recordings on social media.

**Methods:**

This study aimed to answer five research questions. a) Why do individuals use social media? b) Why do people post on social media? c) What types of posts do people share on social media? d) What are the possible psychological reasons that push people to share such occurrences on social media? e) Why do individuals feel the need to record and share dangerous occurrences while under risk or danger? This study was conducted on the basis of a case study design, and interviews were conducted with two psychiatrists, two specialist clinical psychologists, and two sociologists.

**Results:**

After the interviews, the reasons why individuals use social media platforms and post on the said platforms were laid out. It can be argued that the most prominent reason behind individuals’ tendency to post while under risk or threat is isolation and inability to help.

## 1 Introduction

Creating and sharing content in digital environments has become easier with the transition from Web 1.0 to Web 2.0 (Creighton, 2012; Hiremath and Kenchakkanavar, 2016; Lomicka and Lord, 2016; Hackl et al., 2022; Lin, 2023). While it was previously necessary to possess knowledge and expertise to produce content and share it in the digital environment, today, there is no longer a need for a high level of technological or digital literacy to do so (Pangrazio and Sefton-Green, 2021). The produced content attracts more attention when it is posted on social media platforms. Any subject/phenomenon that attracts relatively more individuals is instantly shared with the whole world via social media, finds countless sharers, and is discussed (Tellis et al., 2019; Talwar et al., 2020). This can be attributed to the increase in ownership of smartphones (95.9%) and the easy availability of mobile internet connection (We Are Social, 2023). The same report also states that individuals spend 6 h and 37 min on the internet daily, with 2 h and 31 min of that time being spent on social media. According to Similarweb (2023), the majority of the 20 most popular websites worldwide are social media platforms. Individuals now aspire to be present and post on social media all the time. A life without social media is unimaginable for many (Singh et al., 2019). While the concepts of time and space are being transformed with the digital environment, the line between the watcher and the watched has disappeared, and the individual has become both a watcher and watched (Uluç and Yarcı, 2017; Georgakopoulou, 2021; Oguafor and Nevzat, 2023). One-day posts (stories, statuses) facilitate the use of social networks and enable users to create interesting content based on recent updates rather than events from the past (Davidson-Wall, 2018; Maares et al., 2021). After a point, this situation becomes problematic. Posts where ethical concerns are not at the forefront are now shared on social media. That The social media blurs the boundaries between presence and absence, time and space (Kelly, 2016; Finkler, 2023), control and freedom (Baym and Boyd, 2012; Trepte, 2021), personal and mass communication, private and public (Tombul and Sarı, 2021; Sun et al., 2022), and virtual and real (Floridi, 2014; Levin and Mamlok, 2021). It can be said that this situation makes it easier for individuals to share information that they are hesitant to share in real life, therefore eroding the meaning of privacy and private information (Budak, 2018; Chattopadhyay et al., 2021). Images of people under risk or threat are recorded and shared on social media in different instances, such as parents sharing posts about their children freely (Fox and Hoy, 2019; Verswijvel et al., 2019; Ranzini et al., 2020).

**Figure 1:**
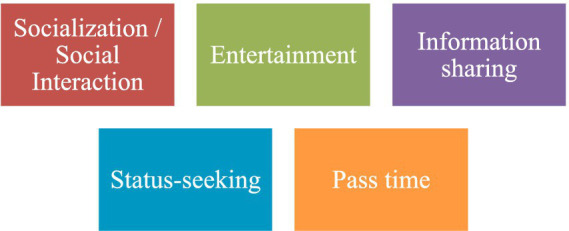
Gratifications for using social media.

By and large, there are various reasons why individuals use the Internet. Subject to change with the content of the accessed websites, the purpose of Internet use can be online communication and socialization with the social circle (Bilgin, 2018), accessing information (Kwon and Wen, 2010; Hazar, 2011; Park and Kim, 2013; Firth et al., 2019), and entertainment (Ryan et al., 2014; Çömlekçi and Başol, 2019) As for social media platforms, the reasons of entertainment and personal gratification stand out (Dunne et al., 2010; Papacharissi and Mendelson, 2011; Ibáñez-Sánchez et al., 2022). According to the Digital 2022 Global Overview Report by We Are Social, among the purposes of using social media are keeping up with the current news and events, finding entertaining content, killing time, keeping up with friends’ lives, and posting (photos, videos, etc.) (We Are Social, 2023). Thanks to online communication channels such as social media platforms, the formation of a relationship between individuals is facilitated, shyness is overcome, and the need for social belonging is met (Young et al., 2017; Moretta and Buodo, 2020). By sharing statuses and photos on social media, individuals promote themselves and meet the need for self-promotion and self-expression (Vilnai-Yavetz and Tifferet, 2015; Tifferet and Vilnai-Yavetz, 2018; Taylor, 2020). It can thus be said that the use of social media by individuals is somewhat related to the desire for gratification and enjoyment (Gan, 2017; Zafar et al., 2020; Scherr and Wang, 2021; Vaterlaus and Winter, 2021). Five different categories related to gratification and enjoyment stand out in the literature (Figure 1).

As seen in Figure 1, socialization or social interaction, entertainment, information sharing, desire to achieve status, and killing time can be listed among the reasons for using social media. Dunne et al. (2010) stated that the primary reason for individuals to use social media is to fulfill the need for socializing. Individuals of all ages use social media platforms actively in order to maintain or expand their friendship networks and to gather or share information (Duggan et al., 2015; Spottswood and Wohn, 2020). The fact that humans are social beings is the underlying reason for socialization and social interaction. Individuals seek to satisfy their need for belonging and interaction with others by chatting and establishing relationships with them (Whiting and Williams, 2013; Alutaybi et al., 2020). Individuals also wonder what their friends do to socialize. Of social media users, 47.6% are curious about and check out what their friends are doing through social media platforms (We Are Social, 2023). Individuals who use social media for entertainment purposes want to enjoy the moment and relieve their day-to-day concerns (Ma et al., 2011; Brailovskaia et al., 2020; Pelletier et al., 2020; Raza et al., 2020). According to a report by We Are Social (2021), the rate of those who use social media to browse through entertaining content is 35%. In terms of information sharing, social media is interactive and allows users to share information via a two-way dialogue (Whiting and Williams, 2013; Aichner et al., 2021). According to a report by We Are Social (2021), the rate of those who use social media for sharing posts is 27.9%. Status-seeking gratification refers to the desire to be correct, therefore strengthening own feelings and morals (Thompson et al., 2019). By sharing posts on social media, individuals contribute to it. When other members of the platform approve of the contribution to the pool of information, it creates the perception of enhancement of the sharer’s social status (Cheung et al., 2011; Verduyn et al., 2020). Among the prominent functions of social media are self-promotion, willingly revealing personal information, and the aspiration to share a much more positive and ideal representation of one’s life (Błachnio et al., 2013; Lupton, 2021; Ugwudike et al., 2023). The pass-time gratification is defined as using social media to kill time and relieve boredom (Whiting and Williams, 2013; Whelan et al., 2020). According to We Are Social (2023), the rate of those who use social media to pass time is 36.3%. Approving or sharing content on social media is about passing leisure time (Kırcaburun et al., 2020). Accordingly, this study aims to determine the sociological and psychological reasons why people record dangerous occurrences where they or other people are under risk or threat and post these recordings on social media. Accordingly, this study aims to answer the following research questions:

RQ1. Why do people use social media?
RQ2. Why do people post on social media?
RQ3. What types of posts do people share on social media?
RQ4. What are the possible psychological reasons that push people to share such occurrences on social media?
RQ5. Why do individuals feel the need to record and share dangerous occurrences while under risk or danger?

This study consists of seven sections. In the following section, the background of the culture of posting on social media will be presented. The third section describes the methodology through which the review processes were conducted. The fourth section presents the results, followed by the fifth section which reports on the research questions’ results. The sixth section presents a discussion of the review, and its conclusion. Finally, the last section presents suggestions to further studies.

## 2 Methods

### 2.1 Research model

Case studies are defined as an in-depth description and exploration of a limited sample (Merriam, 2013) and they aim to reveal why the event occurred in that way and what should be focused on in future studies (Davey, 1990). Case studies have continued to be the preferred research method in this regard (Niemi and Kousa, 2020; Shahzad et al., 2020). Similarly, as the aim of this study is to reveal an existing situation in detail, the case study model was chosen for use in this study.

### 2.2 Participants

The participants of the study are two psychiatrists, two expert clinical psychologists, and two sociologists, who are experts in their fields. Expert psychiatrists, expert clinical psychologists, and sociologists who have conducted studies on social media and the effects of social media have been prioritized while selecting participants. The said experts were contacted via e-mail and informed about the study, and those who accepted to conduct an interview became the participants of the study. The participants were coded as P1, P2, etc. according to the chronological order of interviews.

### 2.3 Data collection and analysis

A semi-structured interview form prepared by the researcher and consisting of five questions was used during data collection. Expert opinions were consulted in order to ensure the validity and reliability of the questions. During the data collection phase, online interviews were held with the mentioned experts, the durations of which varied between 8 and 23 min. The interviews were recorded after obtaining the verbal consent of the participants and the meetings were transcribed afterward. After transcription, the researcher determined the codes, the theme, and the sub-categories.

**Figure 2:**
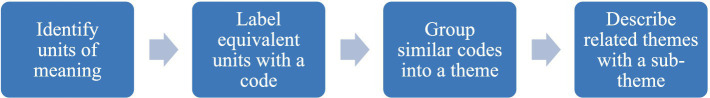
The process of content analysis.

The research findings were obtained through content analysis of the answers given to the questions in the semi-structured interview form. In content analysis, data is categorized into codes, categories, and themes (Miles et al., 2014). Content analysis allows for the analysis of content not only through coding but also by examining themes and subthemes within the content (Graneheim et al., 2017; Lindgren et al., 2020). Yıldırım and Şimşek (2000) emphasize that codes, categories, or themes should be more abstract, general, and inclusive than the concepts obtained in content analysis (Figure 2).

Therefore, the obtained data were first coded, and the codes containing the same expressions were gathered under a common parent theme. After assigning codes and themes, the main themes were determined in line with the purposes described by the questions asked, and the data were grouped accordingly. In order to enhance the reliability of the study by avoiding researcher bias and to keep the internal consistency high, the data were coded by another expert. To enhance the reliability of the research and maintain high internal consistency, the coding process involved having another expert also code the data to protect against the individual influence of the researcher. To strengthen the reliability of the codes determined by both researchers, the agreement formula proposed by Miles et al. (2020) was used (Reliability Formula: Agreement/Agreement + Disagreement * 100). The agreement percentage obtained through this formula was above 85% for all questions. In order for internal consistency to be high, the consensus among the coders is needed (Baltacı, 2017).

## 3 Results

The participants were asked five questions as a part of the study, which investigates the sociological and psychological reasons behind individuals’ urge to post on social media while they or others are under risk or threat. These five questions are (a) Why do people use social media; (b) Why do people post on social media; (c) What types of posts do people share on social media; (d) What are the possible psychological reasons that push people to share such occurrences on social media and (e) Why do individuals feel the need to record and share dangerous occurrences while under risk or danger? According to content analysis, data has been presented in tables as codes, themes, and subthemes. When moving from codes to subthemes, a path is followed from general to specific. This is also explained in Figure 2. In this section, the findings obtained from the answers were addressed along with the respective questions.

### 3.1 Individuals’ purposes for using social media

In this section, findings related to why people use social media are presented. Generally speaking, the reasons why individuals use social media can be divided into groups such as communication, intimidation, commercial, information sharing, presence, and interaction (Table 1).

**Table 1:** Individuals’ purposes for using social media.

Code	Theme	Quotation
Communication	Tracking down old friends	P1: I look my old friends up.
	Contact with people	P2: Umm apart from that, communicating with people they do not know more. P3: To communicate, to establish a social network.
Intimidation		P2: For example, there are some people who disseminate wrong information. Especially in adverse events. About anything. Well, to my understanding, I think it can be to leave a personal mark or awing people or even intimidate them.
Commercial		P4: Some use it for advertising purposes, some use it for commercial purposes. Because now, you know, social media, especially Instagram and YouTube, can be used for commercial purposes as well. Products are sold and relevant posts are shared. P6: Some people use it for the purpose of growing their business. Well, especially young users, umm, they share posts for the sake of sharing, as well as to be of use, to spread knowledge, and announce developments regarding their professions.
Sharing information		P3: To share information. P6: Let us say that I have reached a certain age. I am retired. I have a certain amount of experience and knowledge. In this period of my life, I want to share my knowledge and help people.
Presence	Me too	P5: It is like saying “I am here. Ask me too. My perspective on life and my perception of the world matter. So I’m always there. Me too.” P6: So, to say “I did this, I did that. I am this, I am that.
	Participation in life	P1: When they look for a way to participate in life
	Self-expression	P1: More like a way of self-expression. P5: They feel like they were unnoticed. So, just as all attempts at communication manifest presence, people do the same on social media.
	Self-promotion	P1: It’s about self-promotion. P2: But to present oneself, to awe other people. P4: Self-promotion, popularity are reasons we see frequently. The popularity part is more generally valid for young individuals. P5: They think “Let them use the like button for me, too. Let us see how many people will do so. Am I liked a lot?” They wonder in what way they are liked the most.
Interaction	Followers	P1: They want more followers. P3: It might be to get more likes. P4: And you know, there are people who share a post and get thousands of likes or followers.
	Keeping in the loop	P2: Umm, some get news.
		P1: “I drank coffee there,” “I saw that view”. “I’m at that concert.”

When Table 1 is examined, it can be seen that people undeniably use social media for communication. Social media platforms, which were primarily developed for the purpose of posting photos or messages, now allow people to communicate with each other in visual, audio, and written forms. Even without these features, people could communicate with each other through their posts. Another use of social media is to intimidate others. While sometimes this intimidation takes up the form of bullying, sometimes it is panicking others through fake news. Since social media platforms appeal to people who do not have a sufficient digital literacy level to operate other platforms such as websites, individuals can easily advertise their work on social media. So, social media platforms are also used for commercial purposes. Individuals can advertise their work, just like large companies, which do so through “Influencers.”

Another reason for the use of social media is to share information with others. Some use social media platforms to disseminate information on a certain subject, conveying their fund of knowledge to as many people as possible. Perhaps the most important reason for the use of social media is that it allows individuals to promote themselves, participate in life, and say “me too.” In other words, social media platforms are important in the sense that it enables individuals to manifest their presence. People can see what other people (celebrities) are doing, what they eat, and what they possess on various platforms (one-sided). Social media platforms now provide a medium where average people can promote the activities they do (going around, eating, drinking coffee, taking a vacation, going to the movies, etc.) like the people they follow or other people around them and get the message “I can do it, too/I am doing it” across. The existence of the “Like” button increased users’ efforts to promote their existence. Another reason for the use of social media platforms is the desire of humans, who are social beings, to interact with other people. In addition to interacting with other people, it is important for individuals to have a large number of followers. Because the more the followers, the more the “likes.” People also prioritize not feeling like missing out on something while interacting with others. In traditional news outlets, the act of disseminating news was one-way and in the form of receiving the news. However, on social media platforms, individuals wish to receive news and see posts about people they are curious about quickly and do not want to miss out on the latest developments. Therefore, they turn to social media platforms to receive news.

### 3.2 Individuals’ purposes for sharing posts

In this section, findings related to why people share content on social media are presented. While individuals use social media platforms for these purposes, they also prefer to share posts there, too. The reasons why people share posts on these platforms can be summarized as seen in Table 2.

**Table 2:** Purposes of people sharing posts on social media.

Code	Theme	Quotation
The need to be noticed		P4: If the person is sharing his/her own image, or if he/she is sharing about himself/herself, it may be due to the need to be noticed.
Not missing out on the moment		P1: “Life is going on and I’m missing out on it. I must not miss it.”
Being able to say “Me, too”		P5: “I also have an opinion on this. I want to say it, too”
Comfort		P5: “I can leave a comment on social media and turn off the notification of all replies to that comment. So, I can say whatever I want without letting anyone criticize me.” P6: A lot of things that would not normally be said face to face can be said very easily on social media. This includes many criticisms. Umm, insult, swear, umm threat, humiliation. So, people express themselves more freely on social media.
Storytelling		P1: We used to hear things from others. Like, we would watch other people’s lives. But now, everyone wants to tell their own story. Because a human being is a creature who likes to tell stories, especially about himself.
Responsibility	Oppression	P4: Individuals feel a tremendous amount of pressure for sharing posts, like they have to do it. If they do not, umm, they feel like they did not fulfill their responsibility, so they share posts.
	Notifying the authorities	P4: While sharing posts, they may think, “I should let them know as soon as possible.” “I need to notify the authorities as soon as possible”.
Extrinsic motivation	Living for social media	P1: There are those who share every moment of their lives, 24/7. Of course, umm, at that point, there begins detachment from reality and starting to live for social media.
	Earning money	P1: There really is a group of individuals who lives for social media. Like… Of course, they also make money from it. They gain followers. They gain gratification.

Individuals share posts on social media to fulfill their need to be noticed. Such posts usually are shared in the form of images, mostly containing the individual, too. Individuals may share posts by acting upon the impulse of revealing “I am in these environments like you, see me too.” Another purpose of posting on social media is the desire not to lose the moment. Life is finite and posts can be shared in order to savor specific memories, to save them. People who post on social media do so also in order to be able to say “Me, too.” In other words, people who have an opinion on a subject can express their opinion and answer a question asked on social media. The most important advantage of posing on social media platforms is its convenience. Sometimes individuals recklessly utter statements about others that they would otherwise be unable to tell to their faces; they also can easily make positive or negative comments. While expressing an opinion on a subject, posts can also be closed for comments to prevent anyone from approaching a comment from a critical point of view. Individuals enjoy this convenience while posting on social media. Being social creatures, humans used to watch the stories about the lives of others in traditional media outlets. With social media, however, they found a medium where they can share their own stories. Responsibility is an important factor that pushes people to share posts on social media platforms. This is observed especially in public events. For example, in cases of femicide, posts against it are shared, and individuals think “everyone shares it, I need to share a post about this, too. I have a responsibility.” In some cases, there is an impulse to share posts on social media in order to inform the relevant authorities about an event witnessed somewhere. Extrinsic motivation is perhaps the most influential factor affecting the motivation to share posts on social media platforms. In general, individuals act upon extrinsic motivation factors such as getting likes, gaining followers, or a sense of responsibility. Individuals who share every moment of their lives on these platforms and cannot break away from them are steered by extrinsic motivation. There is another group of individuals who share posts on these platforms because they earn money. Money is also an extrinsic motivation source and posts are shared in order to earn it. There are posts shared on social media platforms for this purpose.

### 3.3 Types of posts

This section includes findings on what type of content people share on social media. The types of content shared for this goal vary (Table 3).

**Table 3:** Types of posts people share on social media.

Code	Quotation
Personal	P4: Personal posts are also shared. I share personal posts myself, too. I also see such posts from other people. P5: They share the things to be noticed. Umm, they can share food, the clothes they wear, where they went, etc. Like, “We were there”.
Perfect life	P1: When you take a look at it, everybody posts the most perfect version of their lives on social media. Like, they do yoga, have brunch, they are having a vacation, and so on. But we do not know the story behind these posts.
Informative	P1: There are also users who share legit information. P3: There are those who share academic posts. P4: Generally, I see posts about mental health, which are related to my field.

What individuals post on social media varies. Personal posts can be regarded as the most prominent type of post on social media. Individuals can share and present themselves on social media with a selfie or an image that features themselves. They share such posts to say “I’m here, too.” The most widespread of posts are those shared in order to promote a perfect, smooth life. Looking at social media platforms, it can be claimed that everyone has a very good life, their income is high, and they lead a happy life. Another type of post shared on social media platforms is informative content. Such posts aim to transfer knowledge from people who possess a certain level of information on a topic to others. Depending on the social media platform, these contents can be in the form of texts or videos. With the live broadcast feature offered by social media platforms, experts in a given field can share their knowledge interactively and in real time. Individuals’ purposes to use social media platforms, why they share posts, and what kind of posts they share can be summarized as so.

### 3.4 Socio-psychological reasons that push people to share on social media

This section includes findings on the psychological reasons that push people to share content on social media. Socio-psychological reasons (Table 4) that push people to share posts on social media are of significant importance. Because individuals use social media platforms voluntarily.

**Table 4:** Socio-psychological reasons that push people to share on social media.

Code	Theme	Quotation
To say “It’s me”		P4: You know, to say “I was the first to share it, people saw it from me.” P5: Individuals want to say “I’m here.”
Responsibility		P4: The individual may feel responsible. So, they feel the obligation to share. They may think “I need to disseminate information.” This may be the state of mind that encourages posting.
Need	Belonging to the majority	P1: In a way, umm, even when you are marginalized, there is actually a need to somehow have a sense of belonging to the majority. The posting kind of meets that need.
	Being Heard	P5: Like, we do not know how to listen to each other. Our education system, unfortunately, does not encourage children to speak. The teacher comes in and gives the lecture. Asks, “Do you have any questions?” But he does it in the last 2 min of the period. If he does it at all. Then he leaves the classroom. It’s the same in the family, too. The parents just ask the child “What did you do today? How was your day, good?” And they do not pay attention to the answer. “Is everything right? Did anything bad happen?,” they ask.
	Staying up to date	P1: What’s trending is the most important. Keeping up with the trends, staying in the loop.
	Being deemed important	P5: From what I see, social media is an environment where people announce their presence. P6: The need to attract attention, to be the focal point
	Relaxation	P1: Individuals, who experience all kinds of negativity in their daily life, may just pick up their phone in the evening and surf around between their different profiles and share a post to relax. Because the opportunities for people to socialize are limited. Really, there are few sports areas, and we hardly spend any time in nature. Not that there’s much nature remaining in the world.

People can share various posts on social media platforms. When we look at the socio-psychological reasons why individuals share such posts, it can be said that the urge of saying “I’m the one” is prominent. They may believe that they should be the first ones to share a post about themselves or a public event. By being the first one to make a comment on such an event, they may want to feel like they are the originator of the information. Or, they may feel the need to announce that they are a part of society, too. Another socio-psychological factor can be claimed to be the feeling of responsibility. Individuals may post due to a sense of responsibility to the environment/society of which they are a part. When we look at the socio-psychological reasons for sharing on social media, it is seen that people share because of a need. These needs can be listed as belonging to the majority, being heard, keeping up with the trends, being deemed important, and relaxing. Being social beings, individuals wish to be included in society and not to be alienated from the majority. Social media platforms offer people an environment where they can make their ideas heard. In everyday life, there are times when people do not listen to each other. However, social media posts are followed with particular attention. So, individuals may use these platforms to be heard. Social media platforms are beginning to replace traditional media and the news now first starts circulating there. Therefore, individuals use social media to get and share the news. The wish to be deemed important is as much a need as the wish to be heard, which social media platforms meet. Social network platforms offer individuals the chance to meet their need to be deemed important through their posts, helping them feel that they are shown the value they deserve. The most prominent socio-psychological reason for posting on or using social media platforms can be claimed to be the need for relaxing. Because in their daily lives, individuals go to work to sustain a living, and worry about themselves and their families. Since opportunities for relaxing in daily life are scarce, social media platforms help individuals relax by browsing around between posts. While individuals generally use social media platforms for these purposes, they may also want to share when they or others are under risk or threat.

### 3.5 Why people share posts when they or others are under risk or threat?

This section includes findings on why people feel the need to record or share the moment when someone else is at risk or in danger. Reasons for posting in these moments Table 5 can be summarized as shown in Figure 2.

**Table 5:** Reasons why individuals share posts on social media platforms when they or others are under risk or threat.

Code	Theme	Quotation
Asking for help		P6: Of course, one of the reasons may be to ask for help. It may be that people try to announce “Something is happening, come and help.”
Raising awareness		P1: Sometimes such posts serve great causes. Like, they may help others gain awareness about a given topic. P6: To draw attention to an occurrence, to raise awareness. Like saying, “Something is happening here, come see.”
Interaction-seeking		P1: You know, such posts also attract a lot of attention. When you record such a happening, you can send it to a news channel, you can share it on your own social media. The high interaction part of it may be attractive. P2: Sharing such posts may make one famous. So, if they put their name in the post they may suddenly get famous, too. P3: Some people, for example, will record such events to post them and get likes. P5: Nowadays, people seek to get likes by having adventures, and doing attractive things more.
Evidence, documentation		P3: Some people may record such moments to document them. They may think, “The least I can do is document it.” And so it happens that there have been instances where such videos helped solve the case. P4: It may also be related to the urge to collect proof. Like, umm, let us say that a crime is being committed. As an eyewitness, individuals may feel the need to record the incident, perhaps due to a feeling of social responsibility or to inform the competent authorities.
Narcissism		P1: “I caught this news.” Here, a narcissistic urge comes into play. Individuals want to be able to say “I recorded that incident” or “I caught that moment”. In other words, it is a situation where we can see the development of the ego at each stage. P3: It could be to have people like them.
A sense of mission		P1: When we look closely, we see people adopt a sense of mission. Like, “I have to record this moment” or “I need to post it”. And it is important that such posts make a tremendous impact.
Isolation	Impartiality	P1: Let us say there is a difficult situation. There is a conflict or something else that’s bad. Some try to be the person who records such an incident. Like, to act as an impartial evaluator of the situation; “I actually have no connection to this happening whatsoever,” a way to escape the consequences. P2: Like, social media can serve as a platform where the intensity of the emotions felt at that moment can be alleviated. If those platforms act as an intermediary, like, if people point to a situation not in real life but through the umm, devices they have, things become easier. Let us say individuals do not scream “Come here! Something is happening!” but announce it via a device, the intensity of the emotions felt at that moment fairly decreases. I do not think people do this on purpose. P4: You know, watching an event live is different from watching it on the screen. So, watching something through a device is similar to watching it on TV. In fact, since individuals are focused on recording the moment, they may feel less overwhelmed by what they are witnessing. P6: When devices go between the individuals and the incidents, the former become alienated from the latter. They pull away, which may make it easier to process the happening. Like, it may serve as a coping mechanism that makes the individual feel that what they are seeing is not real.
Escaping	Responsibility	P1: Umm, the individuals think that by recording the moment but not intervening in it, they are absolved of their moral responsibility. P5: “It’s not my job. Like, let the firefighters put out the fire; let the police catch the criminals; call the ambulance, let the doctor help” mentality may be the reason why, too.
	Problem-solving	P5: Our society does not know how to solve a problem. For example, if there is a violent act, we do not know how we can solve it as individuals. We do not know what to do when there is an inferno, a forest fire.
	Helping	P5: Secondly, individuals think, “What would I be able to do even if I took responsibility?”. This is, for example, a situation we come across very frequently in traffic accidents. A traffic accident occurs, and many citizens rush to the scene of the incident, with all good intentions, but since they do not know how to apply first aid, they break the sufferer’s neck, they hurt his spy and all. Like, what should be done when a tree is on fire? Our people do not know much about it. P6: As the crowd that aims to help grows, everybody passes the responsibility to one another. The responsibility is passed on. Individuals, consciously or unconsciously, adopt the approach of “I do not have to do it; there are so many people here, and one of them will help somehow.”
Security	Self-protection	P2: Because there is a danger to himself. The person is holding a weapon, umm, a club, or another armament. P3: Some are afraid possibly, they refrain from intervening. P4: Well, they may be very clearly scared.

People post on social media when they or others are under risk or threat. It can be held that individuals share such posts primarily for the purpose of asking for help. They cannot intervene in the incident, but record it in order to ask for help from their followers or environment. Another reason for posting in such moments can be to raise awareness. Some incidents may be ignored by traditional media outlets. In such cases, social media posts may make a tremendous impact and help solve issues. For example, such posts can be influential in cases of violence against women or child abuse. The desire to be liked may also be a prominent reason. This desire brings with it attention-seeking, and individuals can increase the number of their followers by sharing such posts on social media, which satisfies the need for receiving attention. In some cases, in order to testify, proof may be needed. In order to have evidence, individuals may record the incident and then post it on social media. Some, due to their excrescent need to be liked, overemphasize their involvement. There may be a desire to say “I saw the incident first; I recorded and shared it” behind not intervening and recording and sharing it as a post. So, it can be said that in such cases, there is a narcissistic underlying motivation. There may also be those who make it their mission to share such content. Also, “Whatsapp Hotlines” were established by traditional media outlets in order to broadcast the images they were not present to record, and people send the content they shoot to those hotlines so that they can be telecast. The theme “isolation” is one of the most important findings of the study. Individuals are used to watching images containing violence on traditional media outlets and in order to maintain this habit, they isolate themselves behind their devices. Therefore, instead of watching incidents with the naked eye, they may need to look at the events behind a screen if they are watching the news on television. The theme of escaping is another significant finding of the study. Individuals may be avoiding responsibility. They may think that it is not their duty to intervene but it is the duty of the relevant officials and therefore avoid responsibility. Another reason for escapism may be the lack of problem-solving skills. In order to intervene in an incident (such as a fight, conflict, or accident), it is necessary to have problem-solving knowledge and skills. In addition, it can be said that the fight-flight-freeze response in the face of a shocking event has been added to the “record and post the moment” response. The same is valid for helping. The ability to help is crucial in the face of an incident and in resolving a problem. If a person does not know how to get a sufferer out of a crashed vehicle, he may instead try to record and share the moment to avoid this responsibility. Individuals, by not intervening in the incident, act upon their instinct for security and safeguarding themselves. Accordingly, they may decide to put a device between themselves and the incident rather than intervening. Another fact is that individuals tend to exhibit unethical behaviors more easily while with others.

## 4 Conclusion and discussion

**Figure 3:**
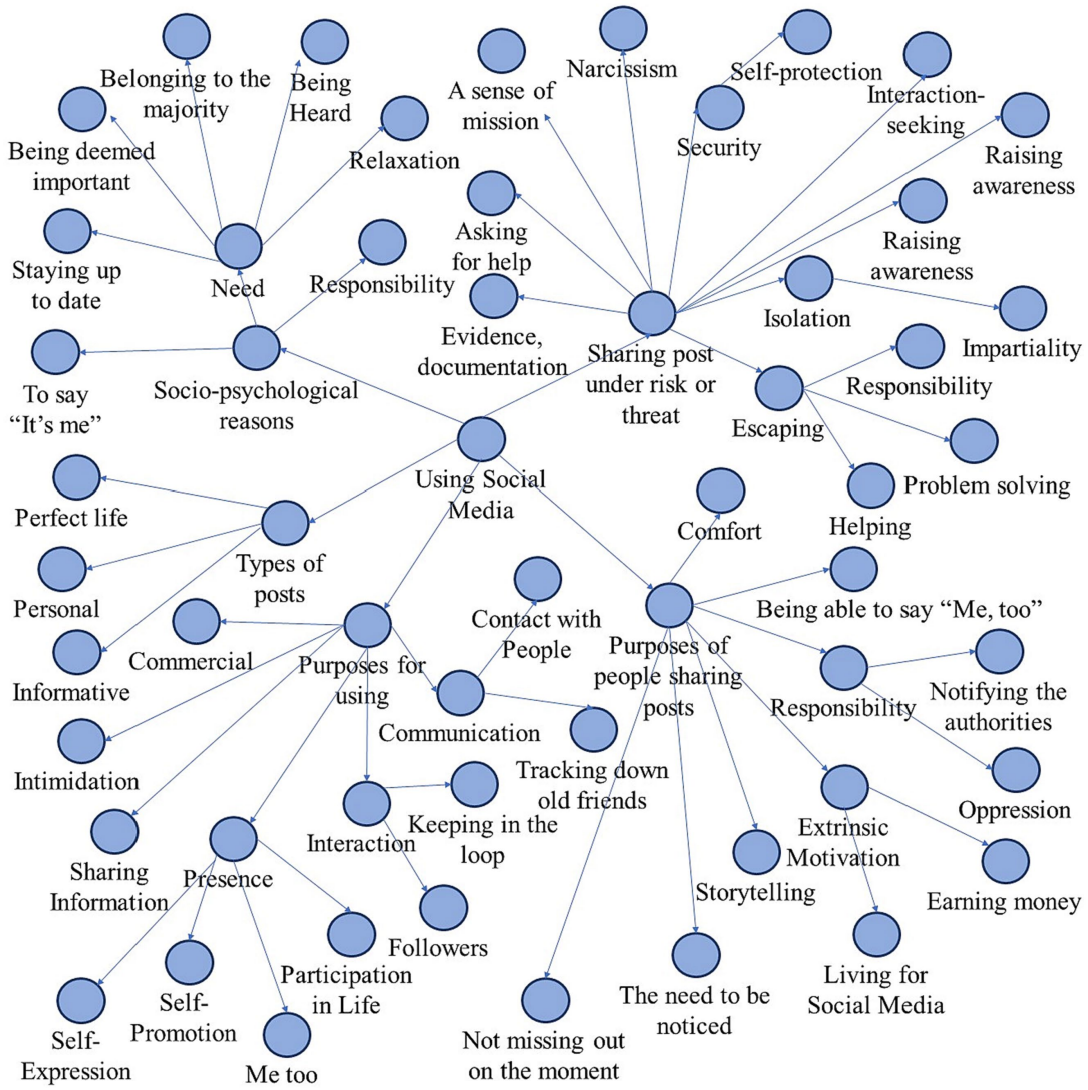
Socio-psychological reasons that influence individuals’ use of social media and their posts.

Different themes and sub-themes were identified in this study, in which the socio-psychological reasons why individuals record and post the moment when they or other people are under risk or threat (Figure 3).

Figure 3 has been organized as a project map in the Nvivo program. The project map is a comprehensive visual aid that assists in discovering the relationships that emerge from the analysis of the data obtained from the study (Mortelmans, 2019). The themes of the purposes of individuals’ social media use can be summarized as communication, intimidation, commercial, information sharing, presence, and interaction. As social beings, humans use social media for communication and interaction. It was stated that social media, which supports participatory communication, is used to express oneself and establish relationships (Sihombing, 2018; Thomas et al., 2020; Guo et al., 2021). People can find their old friends on social media platforms, get in touch with people, follow people they know or do not know, and keep up with the latest developments. Social media platforms are used to inform friends about daily experiences, follow other users’ updates, and participate in long public conversations in discussion groups (Sarmiento et al., 2018). Brailovskaia et al. (2020) also list the purposes of using social media as (a) Search for Information and Inspiration; (b) Search for Social Interaction; (c) Beat of Boredom and Pastimes; (d) Escape from Negative Emotions and (e) Search for Positive Emotions. On the other hand, individuals can share various posts in order to intimidate others, too. Such posts usually do not reflect the truth. Social media can also be used for commercial purposes, as it allows to reach out to many people in a short time. These platforms can also be used to share experiences. There are various studies in the literature on uses and gratifications theory (Katz et al., 1974) that aim to understand the reasons why people use certain media platforms. Introne et al. (2018) stated that some of the gratifications that people attain from the use of social media are related to seeking information, sharing knowledge, entertainment, and communication. Seeking information, maintaining relationships, and gaining peer approval are associated with social media use (Dunne et al., 2010; Vaast and Kaganer, 2013; Nesi et al., 2018; Capone et al., 2020; Liu, 2020; Soroya et al., 2021). Also, it can be said that sharing news and information are other sources of gratification attained from using social media (Thompson et al., 2019). It was stated that people use these platforms in order to have fun and pass time, which is a phenomenon that is not among the findings of the study but has been mentioned (Ha et al., 2013; Kolhar et al., 2021). Social media platforms are also used to relax and escape from reality (Lee et al., 2015; Wang et al., 2015; Young et al., 2017).

The reasons for sharing posts on social media can be listed as the need to be noticed, the desire to not miss out on the moment, the wish to say “me too,” social media offering a comfortable environment, the desire to tell a story, responsibility, and extrinsic motivation. Individuals’ desire to be noticed is not limited to the physical environment, they also try to manifest themselves through their posts on social media. This is why they share posts on social media platforms. Also stated that social interaction and connection, as well as self-promotion, are among the main reasons for the use of social networks such as Facebook (Vilnai-Yavetz and Tifferet, 2015; Hajli et al., 2017; Sheldon and Newman, 2019; Sheldon et al., 2021; Ibáñez-Sánchez et al., 2022). The urge not to miss out on the moment is another prominent reason. Individuals use these platforms in order to show others what they are doing and to see what others are doing. They share posts because social media platforms offer a medium where they can freely express their opinions and say “me too”; want to announce what they do and show their life stories to other people on these platforms. Maitri et al. (2023) state that social media allows individuals to express their views and thoughts freely and that their expressions can reach a wide audience. In some cases, however, individuals, due to extrinsic pressure or the motivation to inform the relevant authorities, may post certain incidents, acting upon their sense of responsibility. Recent research has revealed that when sharing information, individuals do not mind if it is true or not as far as it contains precautionary measures on certain issues (Apuke and Omar, 2020). Another purpose of posting on social media is to act upon extrinsic motivation such as getting likes, gaining followers, or earning money.

People share different types of posts on social media. While some share posts about their personal lives and what they have been up to, some share posts that give the appearance of a perfect life. However, the background of the promoted perfect life is also important. The information presented on social media consists mainly of carefully selected photos intended to portray a flawless self-image and an ideal life (Appel et al., 2016; Wang et al., 2017; Schmuck et al., 2019). The viewership rate of more realistic posts rather than idealized lives is also high on social media (Fardouly and Rapee, 2019). However, users continue to have concerns about editing their photos to appear different from their actual selves. There is another group of users that post informative content. In addition to people who share their own experiences, there are also people who share academic posts. Kwahk and Park (2016) stated that since social media environments facilitate the sharing of information and its dissemination between communities and societies, there is an increase in information sharing. Social networking sites also support the dissemination of information by creating a collaborative environment (Ansari and Khan, 2020), and researchers have begun to utilize social media in sharing information because it facilitates information sharing in different contexts (Al Saifi et al., 2016; Bilgihan et al., 2016; Ahmed et al., 2019). Supporting the findings of the study, it was previously stated that individuals who want to keep their social interactions strong are more likely to disclose information as well as share news, including false ones (Apuke and Omar, 2020). In addition, the sharing of fake news or misinformation is also quite common on social media (Talwar et al., 2019; Islam et al., 2020; Mena, 2020; Apuke and Omar, 2021). However, this phenomenon did not emerge in the study results.

The socio-psychological impact of social media takes into account both social and psychological factors responsible for the changes in the behavior, thoughts, and emotions of young individuals influenced by the real or imaginary presence of others on social media (Afsana, 2020). The socio-psychological reasons why people post on social media are being able to say “it’s me,” the sense of responsibility, and need. Social media platforms are environments where individuals introduce themselves and do so by utilizing their individual capabilities (Thompson et al., 2019; Apuke and Omar, 2021). People sometimes want to be the first to share a post on a certain topic on social media. Or they may try to get the message “I’m here, too” across. Sometimes, individuals may share posts due to the responsibility of alerting the relevant authorities. The most important socio-psychological reasons are that individuals feel the need to belong to the majority, be heard, stay up to date, be deemed important, and relax. To meet these needs, individuals share posts on social media. People with a high motivation to seek status, socialize, and seek information post more frequently on social media (Lee and Ma, 2012; Chen et al., 2018; Islam et al., 2020; Malik et al., 2021). However, individuals find it fun to disseminate information online, to exchange information with others within the confines of a social relationship (Anspach and Carlson, 2020; Oliveira et al., 2020).

Individuals share posts when they or others are under risk or threat, too. Among the reasons for this situation are as seeking help, raising awareness, attention-seeking, collecting evidence, documentation, narcissism, a sense of mission, isolation, escaping, and security. People can record and share incidents for the purpose of asking the authorities for help and to document the incident and gather evidence to submit to the relevant authorities. Some incidents may be underpublicized. In such cases, social media posts may help to raise awareness about those occurrences. As stated by Plume and Slade (2018), individuals may act upon their instinct of spreading news and information, without expecting a reward for their sharing. Al-Rawi (2019) and Weeks and Holbert (2013) also found that people are more likely to share content if it is interesting, useful or emotionally engaging. Apuke and Omar (2021) and Ma and Chan (2014) also argued that some social media users may voluntarily collect and disseminate information without expecting any return. Duffy et al. (2020) state that sharing news on social media is seen as contributing to social integration, and users who engage in this behavior are motivated by the emotional impact of the news, the potential interest for the recipient, and the sender’s intention to provide “recommendation or warning.” While sharing news on social media can build or strengthen relationships, sharing fake news can undermine them. Posts are also shared in order to be the first to announce the incident and increase the number of followers, that is, to attract the attention of other social media users. The number of likes or followers obtained on social media is considered an important personal metric (De Cristofaro et al., 2014). However, attention should also be paid to the following point. If a high number of likes or followers are seen in social media posts, this may also indicate that followers or likes have been purchased (De Vries, 2019). While the number of likes and followers is important, such situations may also occur. These are cases where some make it their mission to record and post the event. Another important finding is the instinct of isolation by putting a device in between the incident and the self, witnessing what is happening as if watching it from social media or traditional media outlets. In order to avoid taking responsibility for solving problems or helping, people tend to record and post images of the moment rather than intervening. During and after risky and dangerous occurrences, individuals also look to protect themselves and ensure their safety. Due to such reasons, more and more images and information from these incidents that should actually remain undisclosed are revealed in the social media environment.

## 5 Limitations

This study has certain limitations. The participants in this study, which investigates the social and psychological reasons behind social media postings, consist of six individuals from three different fields of expertise. Diversifying the fields of expertise may eliminate the limitations of the study in order to access different types of information. Another limitation is the inability to directly access social media users who post under risk and threat.

## 6 Recommendations

Some findings of this study can serve as a guide for future studies. In the literature, there are many data on the purposes for which social media is used [such as sharing stories, fake news or misinformation (Apuke and Omar, 2020, 2021; Duffy et al., 2020; Sihombing and Aninda, 2022; Annabell, 2023; Talwar et.al, 2020)]. However, there are no psychological studies addressing the background of the reasons for why people record dangerous occurrences where they or other people are under risk or threat and post these recordings on social media. This study features expert opinions that can shed light on this subject. In order to support these views and reveal the associated psychological factors, long-term studies can be conducted with the participation of individuals who share posts on social media. Posting on social media should be considered normal. However, the reasons why individuals post when they or others are under risk or threat, which was the main focal point of this study, should be further examined. The researcher considered contacting people who were eyewitnesses of certain incidents and recorded them instead of intervening, such as a person who filmed a femicide and shared it on social media, risked their own life like a war reporter to record and post a gunfight, and filmed their livestock during a forest fire instead of saving them and posting the footage with the caption “my babies, my dears,” and so on. However, these people could not be reached. Studies can be conducted where such people are interviewed to reveal their sociological characteristics as well as their psychological states when they posted the incidents.

## Data availability statement

The datasets presented in this article are not readily available because of confidentiality. Requests to access the datasets should be directed to FY, f.yaman@alparslan.edu.tr.

## Ethics statement

Ethical review and approval was not required for the study on human participants in accordance with the local legislation and institutional requirements. Written informed consent from the participants was not required to participate in this study in accordance with the national legislation and the institutional requirements.

## Author contributions

The author confirms being the sole contributor of this work and has approved it for publication.
